# To the Medical Convention Assembled in Philadelphia in the Month of May, 1847

**Published:** 1847-07

**Authors:** 


### TO THE MEDICAL CONVENTION ASSEMBLED IN PHILADELPHIA IN THE MONTH OF MAY, 1847.

*Report of the Committee on the Organization of the National Medical Association, as ordered by the National Medical Convention held in the city of New York in the month of May,* 1846.

The Medical Convention held in the city of New York, in May last, having resolved, "That it is expedient for the Medical Profession of the United States to institute a National Medical Association," and having appointed a committee of seven, consisting of Drs. John Watson, John Stearns, and F. Campbell Stewart, of the city of New York; A. Stillé, of Philadelphia; N. S. Davis, of Binghamton, N. Y.; W. H. Cogswell, of Plainfield, Conn.; and E. D. Fenner, of New Orleans, "To report a plan of organization for such an association, at a meeting to be held in Philadelphia, on the first Wednesday in May, 1847;" the said committee, after carefully deliberating on the business entrusted to them, beg leave respectfully to report.

### *Plan of Organization for a National Medical Association.*

Whereas, the Medical Convention, held in the city of New York in

May, 1846, have declared it expedient "for the Medical Profession of the United States to institute a National Medical Association;" and,

Inasmuch as an institution so conducted as to give frequent, united and emphatic expression to the views and aims of the Medical Profession in this country, must at all times have a beneficial influence, and supply more efficient means than have hitherto been available here, for cultivating and advancing medical knowledge, for elevating the standard of medical education, for promoting the usefulness, honour, and interests of the Medical Profession; for enlightening and directing public opinion in regard to the duties, responsibilites and requirements of medical men, for exciting and encouraging emulation and concert of action in the profession, and for facilitating and fostering friendly intercourse between those who are engaged in it;—therefore,

Be it resolved, in behalf of the Medical Profession of the United States,—that the members of the Medical Convention held in Philadelphia, in May, 1847, and all others who, in pursuit of the objects above mentioned, are to unite with, or succeed them, constitute a National Medical Association;—and that, for the organization and management of the same, they adopt the following

### REGULATIONS.

### I. TITLE OF THE ASSOCIATION.

This institution shall be known and distinguished by the name and title of "The American Medical Association."

### II. MEMBERS.

The members of this institution shall collectively represent and have cognizance of the common interests of the medical profession in every part of the United States; and shall hold their appointment to membership either as delegates from local institutions, as members by invitation, or as permanent members.

*The Delegates* shall receive the appointment from permanently organized medical societies, medical colleges, hospitals, lunatic asylums, and other permanently organized medical institutions of good standing, in the United States. Each delegate shall hold his appointment for one year, and until another is appointed to succeed him, and shall participate in all the business and affairs of the association.

Each local society shall have the privilege of sending to the association one delegate for every ten of its regular resident members, and one for every additional fraction of more than half of this number. The faculty of every regularly constituted medical college or chartered school of medicine, shall have the privilege of sending two delegates. The professional staff of every chartered or municipal hospital containing a hundred inmates or more, shall have the privilege of sending two delegates; and every other permanently organized medical institution of good standing shall have the privilege of sending one delegate.

*The Members by Invitation* shall consist of practitioners of reputable standing, from sections of the United States not otherwise represented at the meeting. They shall receive their appointment by invitation of the meeting after an introduction from any of the members present, or from any of the absent permanent members. They shall hold their connection with the association until the close of the annual session at which they are received ; and shall be entitled to participate in all its affairs, as in the case of delegates.

*The Permanent Members* shall consist of all those who have served in the capacity of delegates, and of such other members as may receive the appointment by unanimous vote.

Permanent members shall at all times be entitled to attend the meetings, and participate in the affairs of the association, so long as they shall continue to conform to its regulations ; and when not in attendance, they shall be authorized to grant letters of introduction to reputable practitioners of medicine residing in their vicinity, who may wish to participate in the business of the meetings, as provided for members by invitation.

Every member elect, prior to the permanent organization of the annual meeting, or before voting on any question after the meeting has been organized, must sign these regulations, inscribing his name and address in full, specifying in what capacity he attends, and, if a delegate, the title of the institution from which he has received his appointment.

III. MEETINGS.

The regular meetings of the Association shall be held annually, and commence on the second Tuesday of May. The place of meeting shall never be the same for any two years in succession, and shall be determined for each next succeeding year by vote of the Association.

IV. OFFICERS.

The officers of the Association shall be a President, four Vice Presidents, two Secretaries, and a Treasurer. They shall be nominated by a special committee of one member from each state represented at the meeting, and shall be elected by vote on a general ticket. Each officer shall hold his appointment for one year, and until another is elected to succeed him.

*The President* shall preside at the meetings, preserve order and decorum in debate, give a casting vote when necessary, and perform all the other duties that custom and parliamentary usage may require.

*The Vice Presidents*, when called upon, shall assist the President in the performance of his duties, and, during the absence, or at the request of the president, one of them shall officiate in his place.

*The Secretaries* shall record the minutes, and authenticate the proceedings, give due notice of the time and place of each next ensuing annual meeting, and serve as members of the Committee on Publication. The Secretary first in nomination shall also preserve the archives and unpublished transactions of the Association.

*The Treasurer* shall have the immediate charge and management of the funds and property of the Association. He shall be a member of the Committee on Publication, to which committee he shall give bonds for the safe keeping, and proper use, and disposal of his trust. And through the same committee he shall present his accounts, duly authenticated, at every regular meeting.

### V. STANDING COMMITTEES.

The following Standing Committees, each composed of seven members, shall be organized at every annual meeting, for preparing, arranging, and expediting business for each next ensuing year, and for carrying into effect the orders of the Association not otherwise assigned—namely, a Committee on Arrangements, a Committee on Medical Sciences, a Committee on Practical Medicine, a Committee on Surgery, a Committee on Obstetrics, a Committee on Medical Education, a Committee on Medical Literature, and a Committee on Publication.

*The Committee on Arrangements* shall, if no sufficient reasons prevent, be mainly composed of members residing in the place at which the Association is to hold its next annual meeting; and shall be required to provide suitable accommodations for the meeting, to verify and report upon the credentials of membership, to receive and announce all essays and memoirs voluntarily communicated, either by members of the Association, or by others through them, and to determine the order in which such papers are to be read and considered.

*The Committee on Medical Sciences* shall prepare an annual report on the progress of Medical Sciences in America, noticing, as occasion may require, the more important improvements and discoveries in Anatomy, Physiology, Hygiène, General Pathology and Therapeutics, Medical Jurisprudence, Materia Medica, and other branches of natural science, bearing directly on the condition and progress of medical knowledge in America, during the year of their service.

*The Committee on Practical Medicine* shall prepare an annual report on the more important improvements effected in this country in the management of individual diseases; and on the progress of epidemics: referring, as occasion requires, to medical topography, and to the character of prevailing diseases in special localities, or in the United States generally, during the term of their service.

*The Committee on Surgery* shall prepare an annual report on all the important improvements in the management of surgical diseases effected in America during the year.

*The Committee on Obstetrics* shall prepare an annual report on all the important improvements in the Obstetric Art, and in the management of diseases peculiar to women and children, effected in America during the year.

*The Committee on Medical Education* shall prepare an annual report on the general condition of medical education in the United States, in comparison with the state of medical education in other enlightened nations; noticing, as occasion may call for, the courses of instruction, the practical requirements for graduation, the modes of examination for conferring degrees, and the reputed number of pupils and of graduates at the several medical institutions in the United States, during the year:—noticing also the requirements of the United States Army and Navy Boards of Medical Examiners, the legal requirements exacted of medical practitioners in our several states, and all such measures, prospective or established, in reference to medical education and the reputable standing of the profession, as may be deemed worthy of special consideration.

*The Committee on Medical Literature* shall prepare an annual report on the general character of the periodical medical publications of the United States, in reference to the more important articles therein presented to the Profession, on original American medical publications, on medical compilations and compends by American writers, on American reprints of foreign medical works, and on all such measures as may be deemed advisable for encouraging and maintaining a national literature of our own.

*The Committee on Publication*, of which the Secretaries and Treasurer must constitute a part, shall have charge of preparing for the press, and of publishing and distributing such of the proceedings, transactions and memoirs of the Association, as may be ordered to be published. The six members of this committee, who have not the immediate management of the funds, shall also in their own names as agents for the Association, hold the bond of the Treasurer for the faithful execution of his office, and shall annually audit and authenticate his accounts, and present a statement of the same in the annual report of the committee; which report shall also specify the character and cost of the publications of the Association during the year, the number of copies still at the disposal of the meeting, the funds on hand for further operations, and the probable amount of the Assessment to be laid on each member of the Association for covering its annual expenditures.

### VI. FUNDS AND APPROPRIATIONS.

Funds shall be raised by the Association for meeting its curent expenses and awards from year to year; but never with the view of creating a permanent income from investments. Funds may be obtained by an equal assessment of not more than three dollars annually on each of the members; by individual voluntary contributions for specific objects; and by the sale and disposal of publications, or of works prepared for publication.

The funds may be appropriated for defraying the expenses of the annual meetings; for publishing the proceedings, memoirs, and transactions of the association; for enabling the standing committees to fulfil their respective duties, conduct their correspondence, and procure the materials necessary for the completion of their stated annual reports; for the encouragement of scientific investigations, by prizes and awards of merit; and for defraying the expenses incidental to specific investigations under the instruction of the association, where such investigations have been accompanied with an order on the treasurer to supply the funds necessary for carrying them into effect.

VII. PROVISION FOR AMENDMENTS.

No amendment or alteration shall be made in any of these articles, except at the annual meeting next subsequent to that at which such amendment or alteration may have been proposed; and then only by the voice of three-fourths of all the members in attendance.

And, in acknowledgement of having adopted the foregoing propositions, and of our willingness to abide by them, and use our endeavours to carry into effect the objects of this association, as above set forth,—we have hereunto affixed our names.

| NAMES OF MEMBERS. | RESIDENCE. | INSTITUTIONS REPRESENTED. |
|---|---|---|

In connection with the foregoing "Plan of Organization," the committee beg leave further to report the following as one of the ordinances, or by-laws of the proposed association, viz:

THE ORDER OF BUSINESS.

The order of business at the annual meetings of the American Medical Association shall at all times be subject to the vote of three-fourths of all the members in attendance; and until permanently altered, except when for a time suspended, it shall be as follows, viz:

1. The temporary organization of the meeting preparatory to the election of officers.
2. The report of the Committee of Arrangements on the credentials of members; after the latter have registered their names and addresses, and the titles of the institutions which they represent.
3. The calling of the roll.
4. The election of officers.
5. The reading of minutes.
6. The reception of members not present at the opening of the meeting, and the reading of notes from absentees.
7. The reception of members by invitation.
8. The reading and consideration of the stated annual reports from the standing committees.
9. The selection of the next place of annual meeting.
10. The new appointments to fill the standing committees.
11. The choice of permanent members by vote.
12. Resolutions introducing new business, and instructions to the permanent committees.
13. The reading and discussion of voluntary communications introduced through the committee on arrangements.
14. Unfinished and miscellaneous business.
15. Adjournment.

Before bringing this report to a close, the committee beg leave to remark, that in preparing the foregoing "plan of organization," and "ordinance" accompanying it, they have constantly had in view, as worthy of imitation, the plan of organization and order of business adopted without any previous concert of action, by the Medical Convention of 1846.

The plan here presented, differs from the mode of organization adopted by the late Convention, principally in such points as are necessary to give permanency and influence to the proposed association. It is believed to be sufficiently simple and at the same time sufficiently comprehensive and practicable, for organizing the whole of the Medical Profession of the United States into a permanent body; and for carrying into effect all the objects contemplated in the formation of a National Medical Association.

All of which is respectfully submitted.

JOHN WATSON,
JOHN STEARNS,
F. CAMPBELL STEWART,
ALFRED STILLÉ,
N. S. DAVIS,
E. D. JENNER, (by the Chairman.)

